# Recent progress in transition-metal-free functionalization of allenamides

**DOI:** 10.1039/d0ra07119f

**Published:** 2020-10-06

**Authors:** Xiaoxiao Li, Yongchun Liu, Na Ding, Xiaoju Tan, Zhigang Zhao

**Affiliations:** College of Chemistry and Environmental Protection Engineering, Southwest Minzu University Chengdu 610041 People's Republic of China lixiaoxiao.2005@163.com zzg63129@163.com

## Abstract

With their unique reactivity, selectivity, availability and stability, allenamides are receiving increasing attention, and reports on the functionalization of allenamides are rapidly growing in number. In this review, recent developments in transition-metal-free functionalization of allenamides are highlighted. First, developments based on allenamide reactivity are simply introduced. After presenting the advantages of allenamides, recent progress in transition-metal-free functionalization of allenamides is classified and discussed in detail in four parts: chiral phosphoric-acid-catalyzed asymmetric functionalization, iodine-reagent-mediated functionalization, 1,3-H-shift reaction of allenamides, and other metal-free allenamide functionalizations. For the majority of these transformations, plausible mechanisms are presented in detail. The purpose of this review is to provide illustrations of elegant allenamide chemistry, and thereby elicit further interest from the synthetic community to develop novel allenamide methodology.

## Abstract 2

Recent progress in transition-metal-free functionalization of allenamides is reviewed, providing a powerful approach to rapidly access complex molecules.

## Graphical abstract

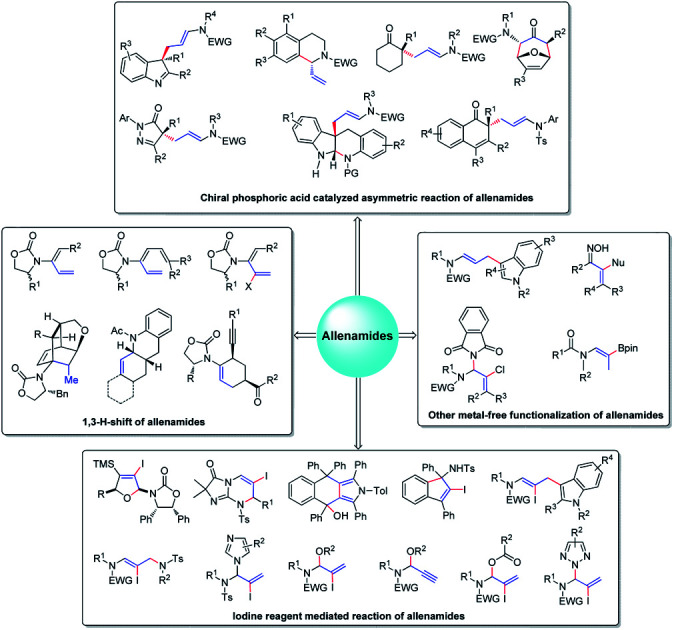

## 1. Introduction

**Scheme 1:**
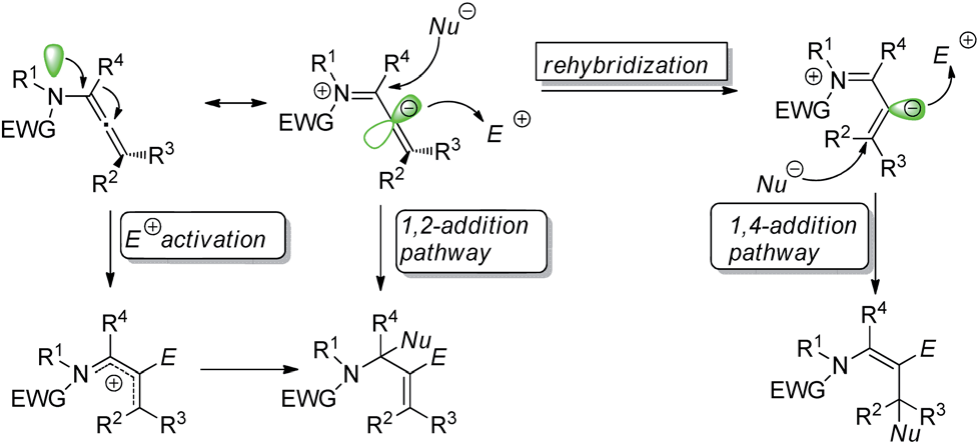
Reaction model of allenamides.

Regio- and stereo-controlled functionalization of carbon–carbon double bonds has enormous potential in organic synthesis.^1^ Allenamides have been widely investigated and utilized as one of the most powerful and versatile building blocks in the field of organic synthesis since the first documentation of their synthesis and characterization in 1968 by Viehe.^2^ The π-donating ability of nitrogen atoms renders allenamides more electron-rich than simple allenes, which predisposes them to electrophilic activation. An electronic bias can be exerted through delocalization of the nitrogen lone pair toward the allenic moiety, as demonstrated in the allenamide resonance form. Accordingly, highly regioselective transformations can be achieved by the consecutive addition of electrophiles and nucleophiles (Scheme 1).

With their unique reactivity, selectivity, availability and stability, allenamide functionalization is being increasingly investigated. While a recent review of the flexible reactivity of allenamides in transition-metal-catalyzed functionalization reactions has been published,^3^ the present review predominantly summarizes progress in transition-metal-free allenamide functionalization in recent years.

## 2. Chiral phosphoric-acid-catalyzed functionalization of allenamides

The hydrogen bond activation mode is recognized as one of the most powerful strategies in the field of metal-free asymmetric manipulation of heteroatom-based organic functional groups and anions.^4^ Chiral phosphoric acid (CPA)-catalyzed synthesis of value added building blocks, discussed in this section, is continuously expanding within the asymmetric synthesis context.^5^

**Scheme 2:**
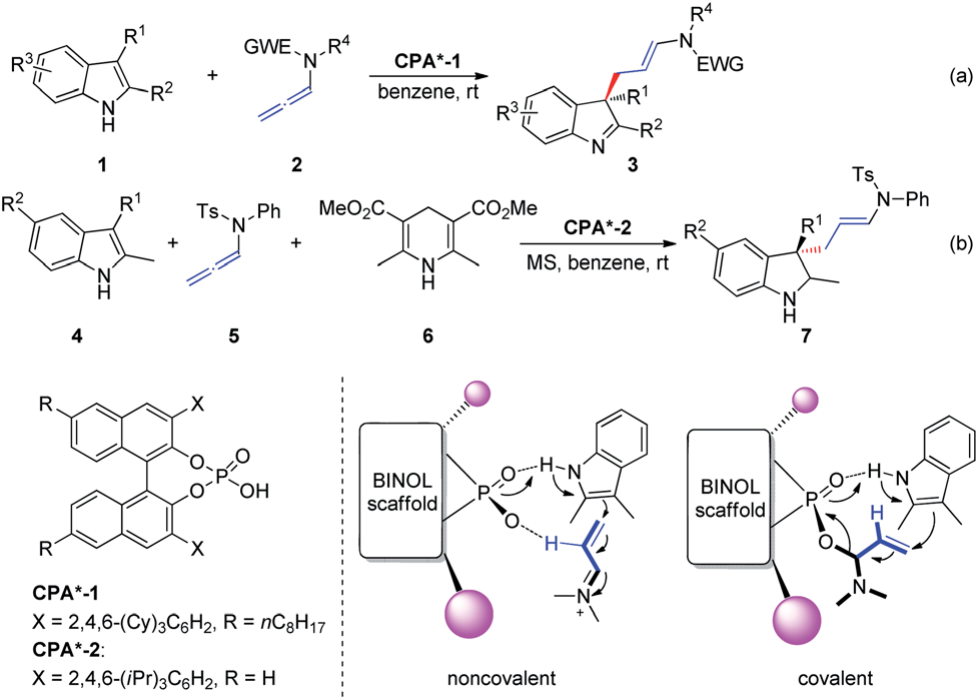
Chiral phosphoric-acid-catalyzed asymmetric dearomatization of indoles.

In 2014, Bandini *et al.*^6^ reported the first effective and unprecedented chiral BINOL phosphoric-acid-catalyzed (1–10 mol%) dearomatization of indoles 1 occurring *via* electrophilic activation of allenamides 2 (ee up to 94%). The realization of this transformation resulted in the direct synthesis of densely functionalized enantiomerically enriched 3,3-disubstituted indolenine cores 3 featuring an all-carbon quaternary stereogenic center at the C3 position (Scheme 2a). Moreover, the authors extended this methodology to the preparation of enantiomerically enriched 3,3-disubstituted indolines 7*via* a Brønsted-acid-catalyzed one-pot dearomatization/hydrogenation transfer sequence entailing a three-component reaction between indole 4, allenamide 5, and Hantzsch ester 6 (Scheme 2b). The authors proposed two possible activation modes: non-covalent and covalent CPA-allenamide interactions. Subsequently, the reaction mechanism was investigated by means of density functional theory calculations and electrospray ionization mass spectrometry analysis.^7^ The first step of the process (rate determining step) was the formation of a covalent adduct between the allenamide and chiral organo-promoter. The resulting chiral α-amino allylic phosphate undergoes dearomative condensation with indoles. In the first step, the indole moiety remains bonded to the catalyst through strong hydrogen bonding.

**Scheme 3:**
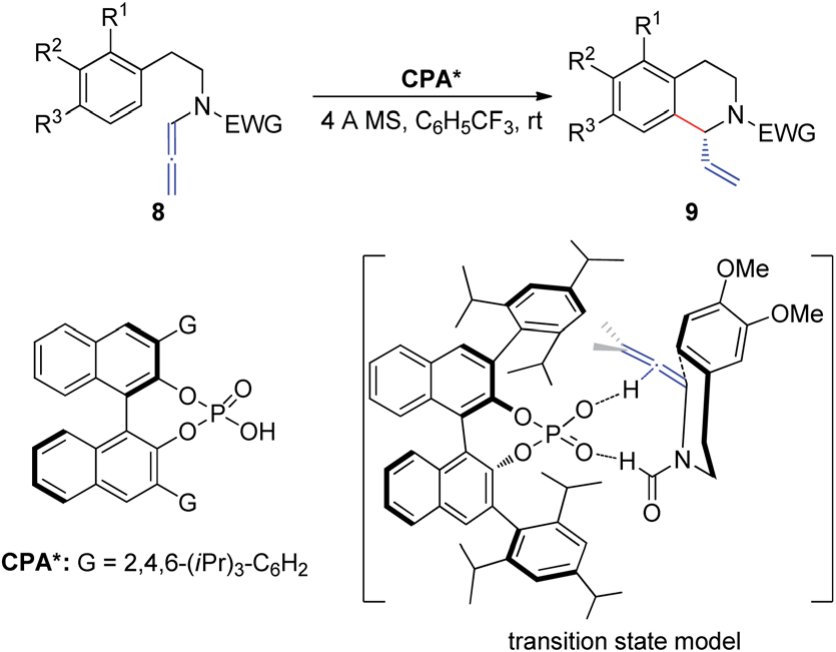
Chiral phosphoric-acid-catalyzed asymmetric cyclization of allenamides.

In 2015, Cozzi *et al.*^8^ reported the cyclization of allenamides 8 to 1-vinyl tetrahydroisoquinolines 9 in the presence of CPAs (Scheme 3). In this reaction, the elusive and relatively unstable iminium ion derived from acrylaldehyde is generated *in situ* and this electrophilic intermediate can engage in stereoselective intramolecular Friedel–Crafts-type allylic alkylation with electron-rich aromatic rings. In particular, given the importance of the formyl group, the authors assumed that the recognition and high enantiomeric excess obtained in the reaction were governed by the hydrogen bonding between the catalyst and the hydrogen atom of the formyl group.

**Scheme 4:**
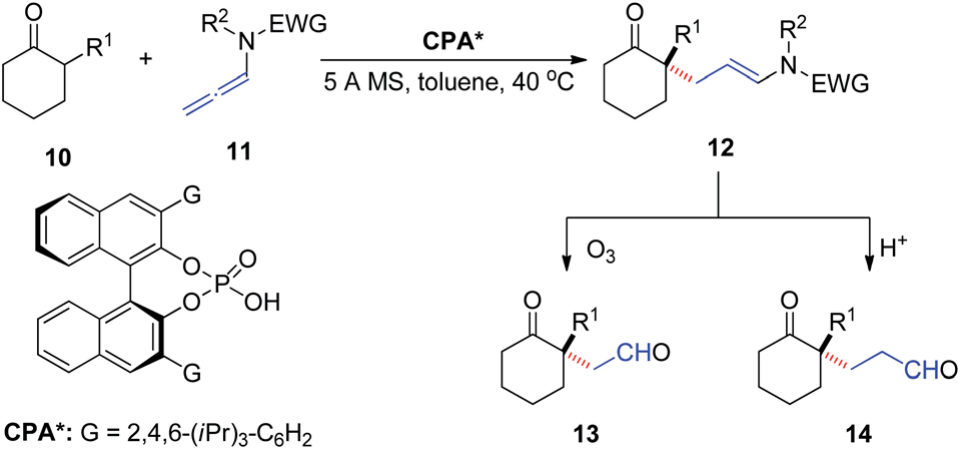
Chiral phosphoric-acid-catalyzed asymmetric addition of unactivated a-branched cyclic ketones to allenamides.

In 2016, Toste *et al.*^9^ reported the asymmetric addition of unactivated α-branched cyclic ketones 10 to allenamides 11 catalyzed by a CPA, to generate an all-carbon quaternary stereocenter 12 with high enantioselectivity (Scheme 4). The reaction exhibited a broad substrate scope, tolerating various aryl, alkenyl, alkynyl, and alkyl substituents as well as cyclohexanone modification. The products could be readily transformed into their corresponding 1,4- and 1,5-ketoaldehyde derivatives 13 and 14, respectively, both of which are important building blocks in organic synthesis.

**Scheme 5:**
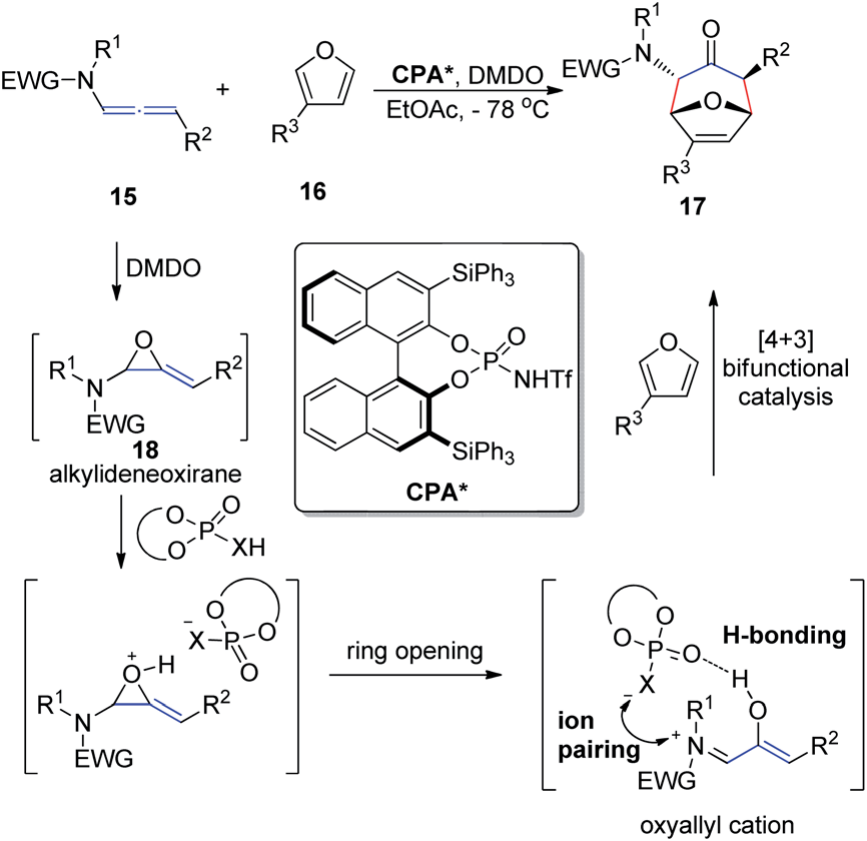
Enantioselective [4 + 3] cycloaddition catalyzed by chiral *N*-trifluoromethanesulfonyl phosphoramides.

In 2017, Vicario *et al.*^10^ reported that BINOL-based *N*-trifluoromethanesulfonyl phosphoramides catalyze the enantioselective [4 + 3] cycloaddition between furans 16 and oxyallyl cations 18, the latter being generated *in situ* from allenamide 15 oxidation (Scheme 5). This method provides a direct and facile access to a wide range of potentially valuable seven-membered rings 17 in a highly regio-, diastereo-, and enantioselective fashion. The reaction relies on the potential of the conjugate base of the *N*-trifluoromethanesulfonyl phosphoramide catalyst to engage in a bifunctional mode of activation, which combines hydrogen bonding with electrostatic interactions by ion pairing with the oxyallyl cation dienophile. This combination enables efficient chirality transfer to the newly formed stereocenters. Moreover, this catalyst system displays a remarkably wide substrate scope with respect to both the furan and allenamide coupling partners, and the excellent performance of γ-substituted allenamides as oxyallyl cation precursors is highlighted.

**Scheme 6:**
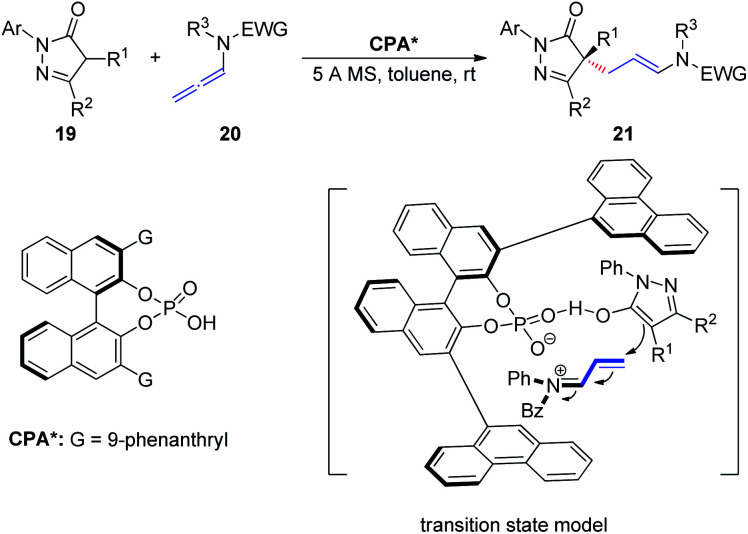
Chiral phosphoric-acid-catalyzed asymmetric addition of pyrazolones to allenamides.

In 2018, Wang *et al.*^11^ described an asymmetric allylic alkylation of pyrazolones *via* CPA-catalyzed asymmetric addition of pyrazolones 19 to allenamides 20 (Scheme 6). The room temperature reaction generates a chiral quaternary stereocenter in high yield and with good enantioselectivity and exhibits a broad substrate scope. Mechanistically, the chiral ion pair generated from protonation of the allenamide by the CPA dictates the enantioinduction of the asymmetric addition process together with an additional hydrogen bonding interaction.

**Scheme 7:**
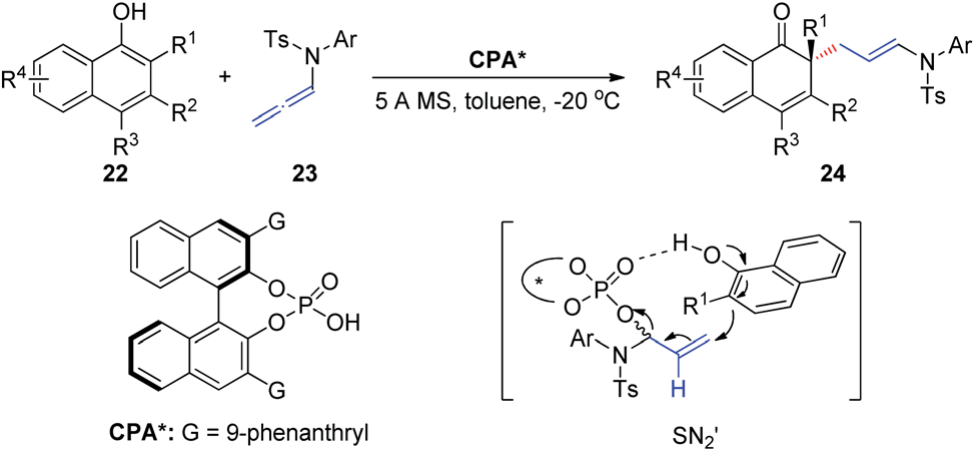
Chiral phosphoric-acid-catalyzed asymmetric dearomatization of α-naphthols.

In 2019, Shao *et al.*^12^ reported the first chiral phosphoric-acid-catalyzed asymmetric intermolecular C–C bond-forming dearomatization of α-naphthols 22 (Scheme 7). This method is also applicable to β-naphthols. The transformation proceeds with high chemo- and enantioselectivity *via* an allylic substitution reaction and provides enantioenriched α- and β-naphthalenones bearing an all-carbon quaternary center. Notably, two distinct possible reaction mechanisms can be considered; the first involves a concerted asynchronous S_N_2-like displacement, while the second entails participation of an α,β-unsaturated iminium ion formed upon protonation by the allenamide.

**Scheme 8:**
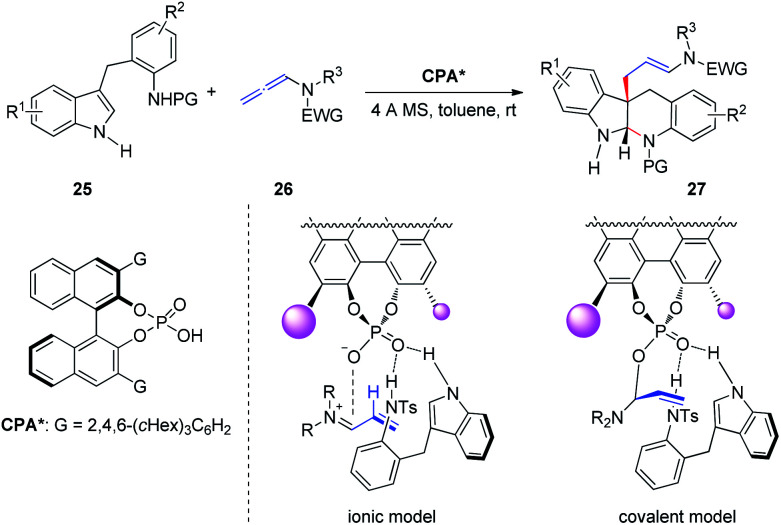
Chiral phosphoric-acid-catalyzed asymmetric dearomative cyclization of homotryptamine derivatives.

Subsequently, Shin *et al.*^13^ reported the CPA-catalyzed asymmetric dearomative cyclization of homotryptamine derivatives, furnishing enantioenriched indolo[2,3-*b*]quinolone scaffolds 27 in up to 99% ee (Scheme 8). The authors proposed two possible stereochemical models: the basic site of the phosphate in CPA activates homotryptamines 25*via* dual hydrogen bonding with the indole and aniline in 25, whereas activation of allenamides 26 by protonation at C2 would form an α,β-unsaturated iminium ion, setting the stage for conjugate addition by the indole moiety of 25 (ionic model). Alternatively, basic phosphate may form a covalent adduct with the iminium to generate an allylphosphate aminal (covalent model), followed by S_N_2 attack by the indole C3 carbon. The authors indicated that the covalent model is more favorable than the ionic model based on Bandini's report.^6^

## 3. Iodine-reagent-mediated functionalization of allenamides

**Scheme 9:**
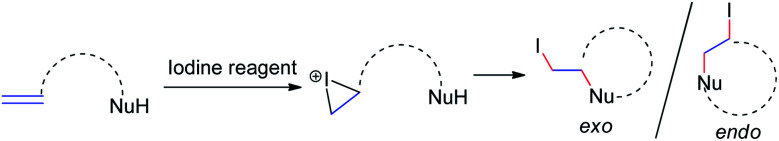
The reaction model between iodine electrophiles and carbon–carbon double bonds.

Iodine-containing reagents have received considerable attention as they are inexpensive, nontoxic, and readily available electrophiles that interact with double bonds.^14^ The first step in this type of reaction is the interaction between the iodine electrophile and the π-system of the alkene to generate iodiranium intermediates, which subsequently undergo an addition or a cyclization reaction, depending on the stereochemistry and ring size formed, in either *exo* or *endo* fashion, as shown in Scheme 9. This segment reviews recent developments in intramolecular cyclizations and intermolecular nucleophilic additions of allenamides mediated by iodine reagents.

**Scheme 10:**
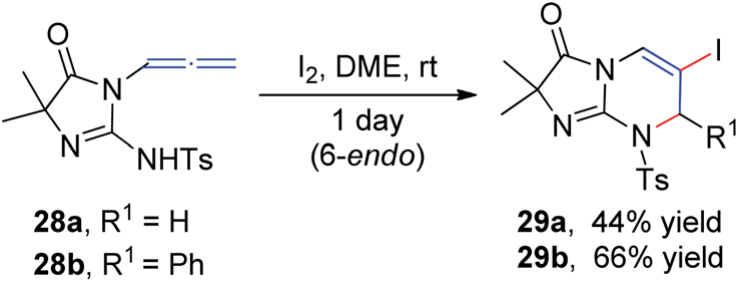
Iodine-mediated intramolecular cyclization of 3-allenyl-1-imidazolinones.

In 1996, Noguchi *et al.*^15^ reported the iodine-mediated intramolecular cyclization of *N*-3-allenyl-1-imidazolinones 28 to give 6-*endo* cyclization products 29a and 29b in 46% and 66% yields, respectively (Scheme 10).

**Scheme 11:**
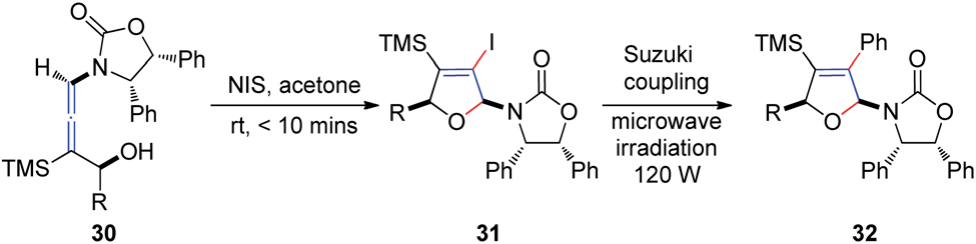
*N*-Iodosuccinimide-mediated cyclization of chiral γ-substituted allenamides.

Subsequently, Hegedus *et al.*^16^ developed an *N*-iodosuccinimide-mediated cyclization of chiral γ-substituted allenamides 30, yielding *cis*-iododihydrofurans 31 with retention of stereochemistry, achieved within 10 min (Scheme 11). The vinyl iodide functionality present in dihydrofurans can be further reacted with phenylboronic acid under Suzuki coupling conditions, providing coupling product 32 in 89% yield under microwave irradiation.

**Scheme 12:**
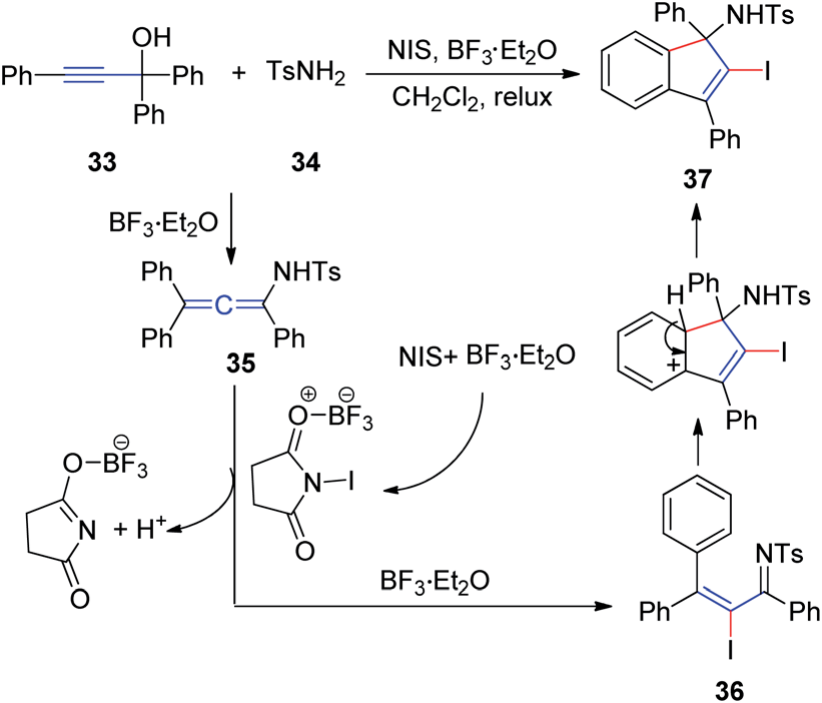
BF_3_·Et_2_O-catalyzed tandem reaction of propargyl alcohol, sulfonamide, and NIS.

In 2011, Wang *et al.*^17^ developed an efficient method to generate *N*-(2-iodoinden-1-yl)arenesulfonamides *via* a BF_3_·Et_2_O catalyzed tandem reaction of propargyl alcohol 33, sulfonamide 34, and NIS (Scheme 12). Allenesulfonamide 35 is postulated to be a key intermediate for this tandem transformation. Mechanistically, propargyl alcohol and *p*-toluenesulfonamide are first converted to the key allenesulfonamide intermediate under BF_3_·Et_2_O catalytic conditions. Meanwhile, in the presence of BF_3_·Et_2_O, NIS is activated to an iodonium species, which activates the allenesulfonamide *in situ* to afford α-iodo-α,β-unsaturated sulfonamide 36. Sulfonamide 36 was subsequently transformed into the final product 37*via* an intramolecular Friedel–Crafts reaction promoted by BF_3_·Et_2_O.

**Scheme 13:**
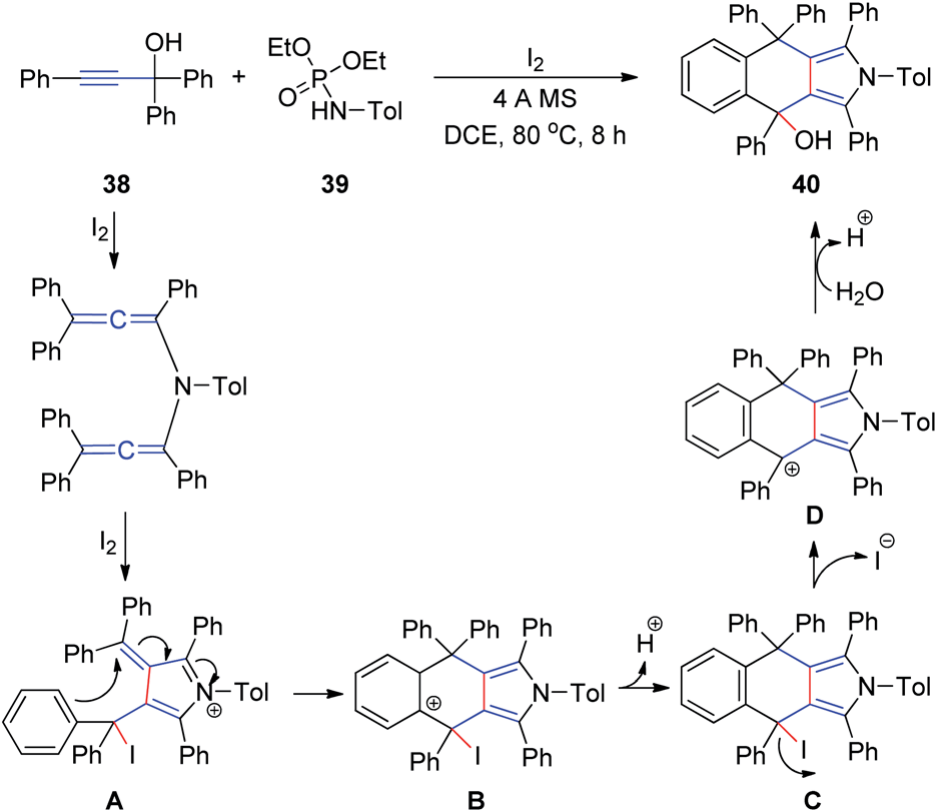
Synthesis of 4,9-dihydro-2*H*-benzo[*f*]isoindoles from propargyl alcohols and phosphoramides in the presence of iodine.

At a later stage, the same group^18^ developed a new method for the synthesis of 4,9-dihydro-2*H*-benzo[*f*]isoindoles 40 from propargyl alcohols 38 and phosphoramides 39 in the presence of iodine in a single step (Scheme 13). First, a diallenamide is formed from propargyl alcohol and phosphoramide in the presence of iodine. Further iodination induces the first cyclization to give compound A. The phenyl ring of A then immediately attacks the α,β-unsaturated iminium intramolecularly to execute the second cyclization and deliver B. Aromatization of the resulting C generates D, which is hydrated to afford 40.

**Scheme 14:**
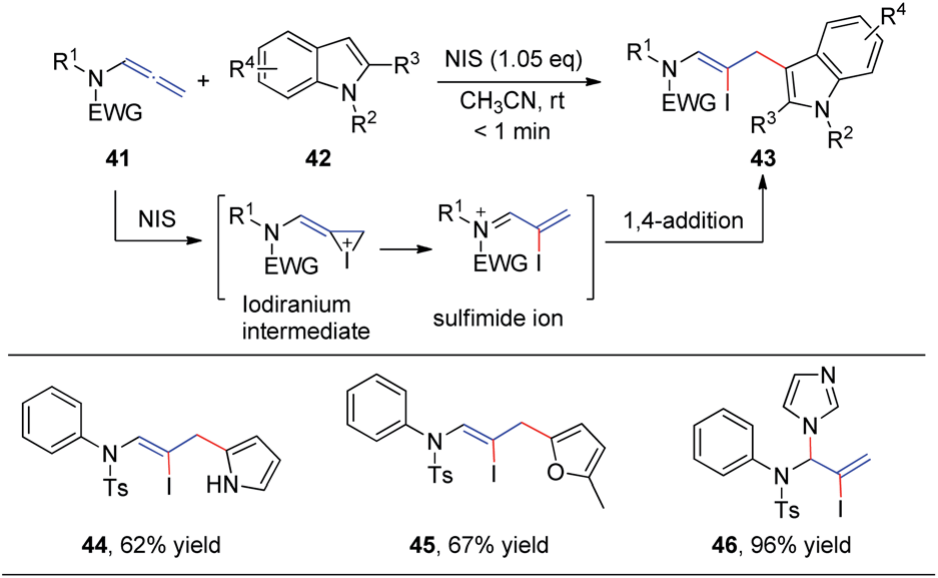
NIS-mediated intermolecular iodofunctionalization of allenamides with indoles, pyrrole, and furan.

In 2016, our group^19^ reported the first *N*-iodosuccinimide-mediated intermolecular iodofunctionalization of allenamides 41 with indoles, pyrroles, and furans, affording the desired 1,4-addition products 43, 44, and 45, respectively, in good yields under mild conditions (Scheme 14). Moreover, when imidazole was used as the nucleophile, the corresponding 1,2-addition product 46 was obtained in good yield. The reaction mechanism involves an iodiranium intermediate, which undergoes a decyclization reaction through the delocalization of the nitrogen lone pair toward the alkene to form the key conjugated sulfimide ion species intermediate. Subsequently, the conjugated sulfimide ion undergoes regioselective addition to give the desired product 43.

**Scheme 15:**
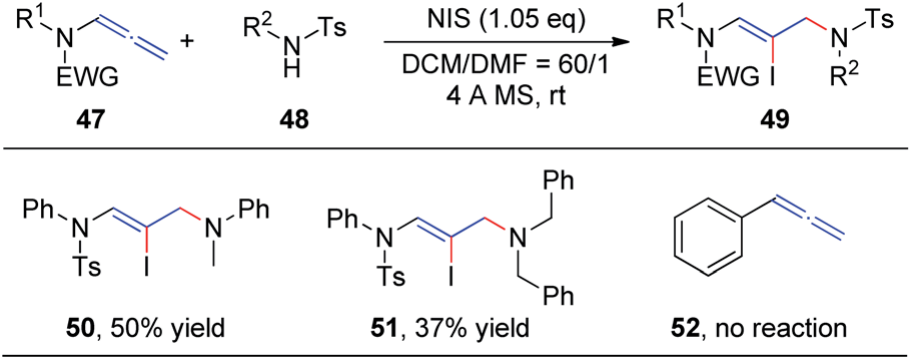
NIS-mediated iodoamination of allenamides.

In our preceding report, we found that the degradation product 4-methyl-*N*-phenylbenzenesulfonamide 48 could also be used as a nucleophile to obtain 1,4-addition products, iodine-substituted allylamino *Z*-enamides 49 (Scheme 15). Therefore, we further studied the NIS-mediated iodoamination of allenamides 47 with sulfonamides 48.^20^ These reactions proceed rapidly and tolerate a broad range of substrates. Moreover, *N*-methylaniline and dibenzylamine likewise reacted efficiently with allenamides to obtain the desired products 50 and 51, respectively, in moderate yields, while unfunctionalized allene 52 could not afford the iodoamination product.

**Scheme 16:**
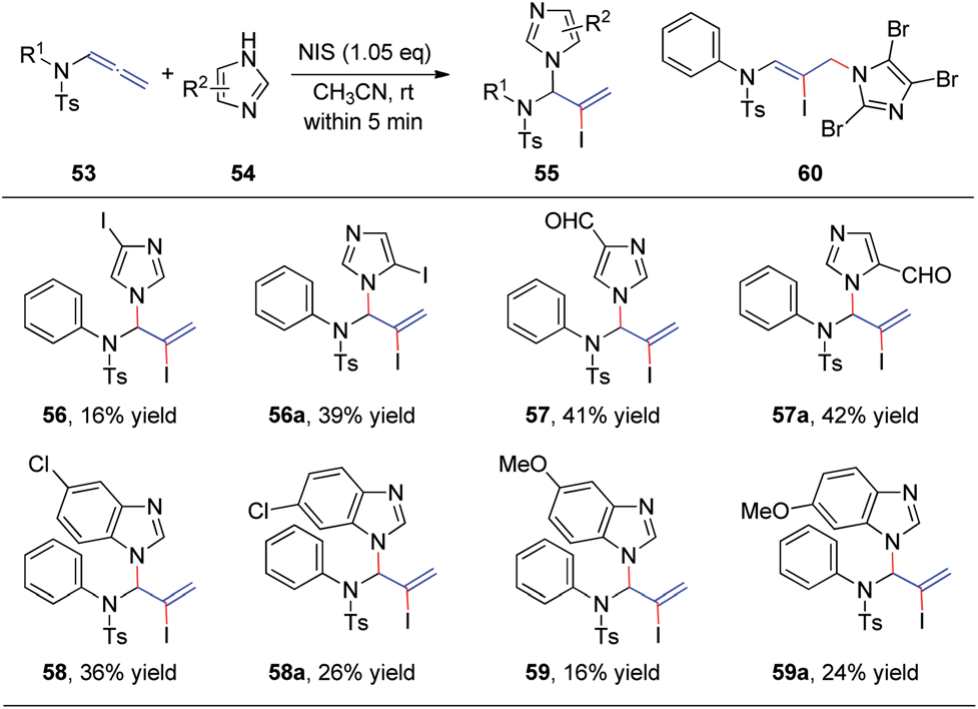
Regioselective iodoamination of allenamides to generate imidazole heterocycles.

Subsequently, we further extended this regioselective iodoamination of allenamides 53 to imidazole heterocycles 54 (Scheme 16).^21^ It is noteworthy that the regioselectivity of this iodoamination appears to differ from that observed in our earlier report^19^ involving sulfonamides, to obtain the 1,2-adduct. The facile reaction is regioselective and tolerant of a broad range of imidazole and benzimidazole derivatives. The key intermediate is a conjugated sulfimide ion species that undergoes nucleophilic attack by imidazole to form the 1,2-adduct 55. Moreover, mixtures of *N*^1^- and *N*^3^-substituted isomers 56/56a–59/59a were obtained using asymmetrically substituted imidazoles, such as 4-iodoimidazole, imidazole-3-carbaldehyde, 5-chloro-1*H*-benzimidazole, and 5-methoxy-1*H*-benzimidazole. In addition, trisubstituted imidazole produced the 1,4-adduct 60 exclusively, because of steric hindrance.

**Scheme 17:**
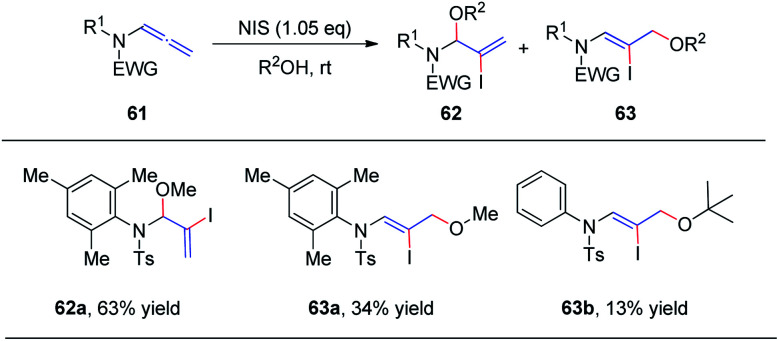
*N*-Iodosuccinimide-mediated regioselective 1,2-additions of alcohols to allenamides.

In 2018, we reported the *N*-iodosuccinimide-mediated regioselective 1,2-addition of alcohols to allenamides 61 for the assembly of a series of N, O-aminals 62 (Scheme 17).^22^ These novel reactions proceed rapidly and exhibit a broad substrate scope for a variety of allenamides. It is noteworthy that the alcohol serves as both the solvent and nucleophile in this transformation. Moreover, when trimethylphenyl allenamide was used as the reactant, the 1,2-adduct 62 was obtained in 63% yield, together with the 1,4-adduct 63a (34% yield); and when *tert*-butanol was employed as the nucleophile and solvent, 1,4-addition product 63b was isolated in 13% yield as the major product, with both of these experimental results possibly arising from increased steric hindrance.

**Scheme 18:**
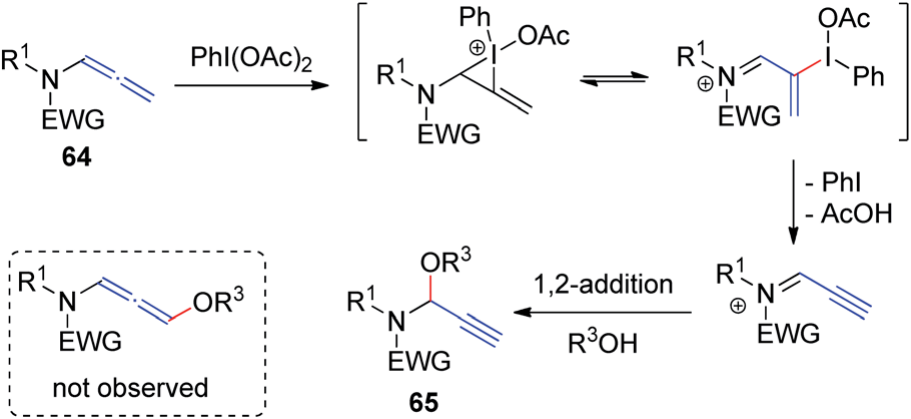
Synthesis of propargylic N, O-acetals *via* hypervalent-iodine-mediated activation of allenamides in alcohols.

Yu *et al.*^23^ recently demonstrated hypervalent iodine-mediated activation of allenamides 64 in alcohols to obtain propargylic N, O-acetals 65 in high yields with excellent regioselectivity (Scheme 18). By using PhI(OAc)_2_ as the oxidant and an alcohol as both nucleophile and solvent, allenamides were converted to propargylic N, O-acetals 65*via* 1,2-addition of alcohol to the sulfimide ion intermediate; 1,4-adducts were not detected.

**Scheme 19:**
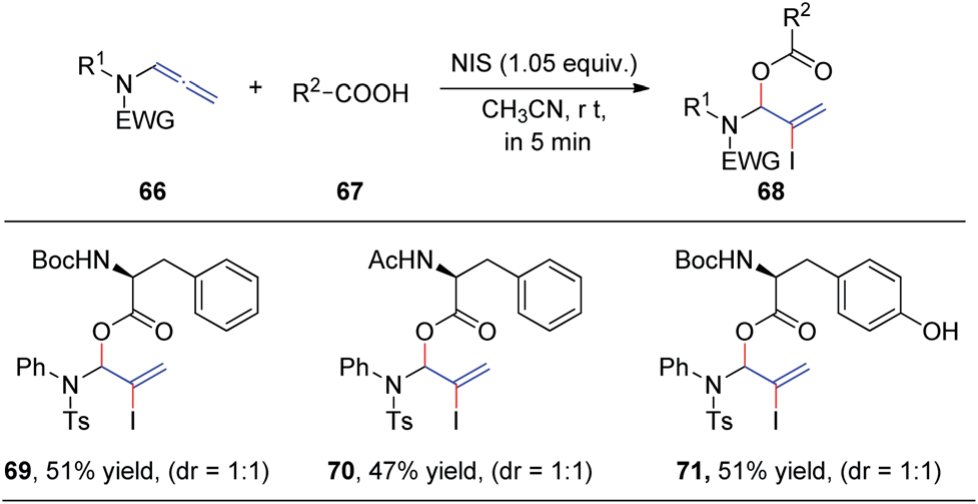
*N*-Iodosuccinimide-mediated intermolecular addition of carboxylic acids to allenamides.

In 2019, we demonstrated the first intermolecular addition of carboxylic acids 67 to the proximal carbon of allenamides 66 toward the regioselective formation of highly useful branched allylic esters 68 by employing *N*-iodosuccinimide (Scheme 19).^24^ The reaction proceeded rapidly and displayed a broad substrate scope, providing an efficient and practical protocol for the synthesis of branched allylic esters. Notably, protected amino acids *N*-Boc–l-Phe, *N*-Ac–l-Phe, and *N*-Boc–l-Tyr were tolerated under the reaction conditions and afforded allylic amino acid esters 69, 70, and 71, respectively, in moderate yields and 1 : 1 dr.

**Scheme 20:**
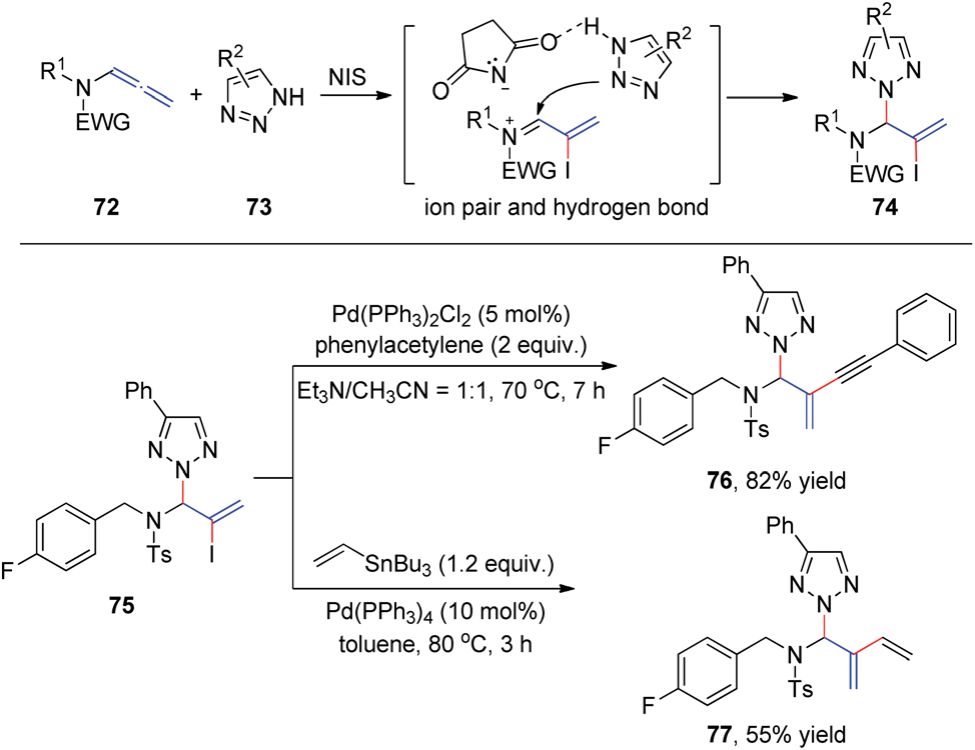
*N*-Iodosuccinimide mediated *N*^2^-allylation of triazoles with allenamides.

Subsequently, we developed a new method for the synthesis of *N*^2^-allyl-1,2,3-triazoles *via* NIS-mediated allylation of allenamides 72 with mono- and unsubstituted *N*H-1,2,3-triazoles and benzotriazole 73 (Scheme 20).^25^ The reaction is facile and highly regioselective. The regioselectivity may be induced by the ionic pair composed of a σ-complex and the conjugate base of the imide through hydrogen bonding between the conjugate base and *N*H-1,2,3-triazole. To further demonstrate the utility of this protocol, product 75 was reacted with phenyl acetylene and vinyl tributylstannane under Sonagashira^26^ and Stille^27^ cross-coupling conditions, and the corresponding coupling products 76 and 77 were isolated in 82% and 55% yields, respectively.

## 4. 1,3-H-shift reaction of allenamides

**Scheme 21:**
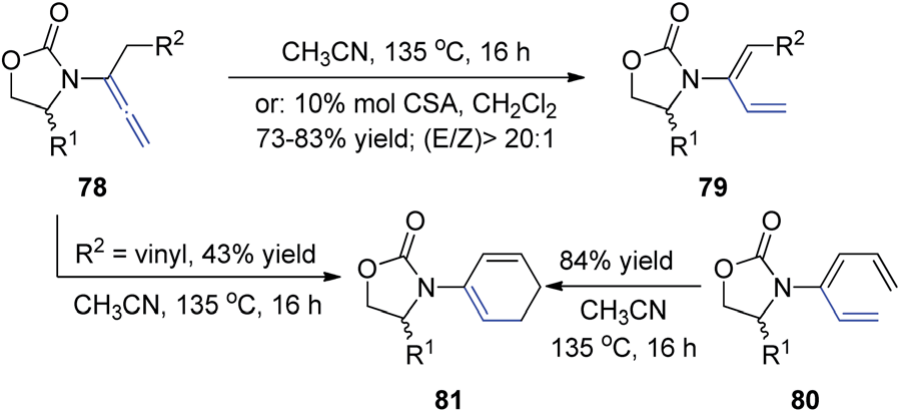
Synthesis of 2-amido-dienes *via* 1,3-H-shift of allenamides.

In 2009, Hsung *et al.*^28^ described the regio- and stereoselective isomerization of allenamides under thermal or acid-promoted conditions, leading to the preparation of *de novo* 2-amido-dienes and a tandem isomerization-6π-electron electrocyclic ring closure (Scheme 21). This 1,3-H shift was found to be highly regio- and stereoselective, as products 79 were obtained in >20 : 1 *E*/*Z* ratios. The excellent *E*-selectivity provided a platform for a pericyclic transformation, as allenamide 78 underwent isomerization to give 3-amido-triene 80 in 89% yield. Subsequently, a thermal 6π-electron electrocyclic ring closure of 80 gave cyclic diene 81. Alternatively, cyclic diene 82 could also be obtained directly from allenamide 80 under thermal conditions in a tandem sequence, albeit in a lower overall yield.

**Scheme 22:**
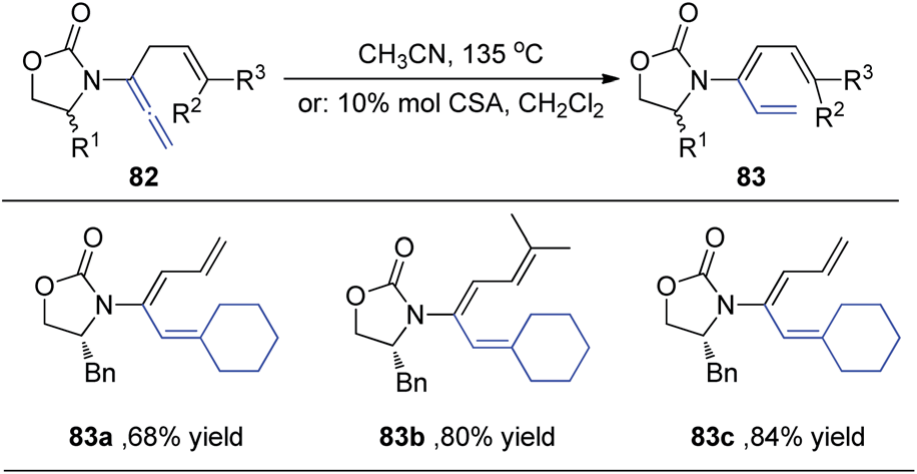
The synthesis of 3-amido-trienes through 1,3-H-shift of allenamides.

Hsung *et al.*^29^ subsequently expanded the substrate scope of the 1,3-H shift reaction for the preparation of 3-amido-trienes 83. Such 1,3-hydrogen shifts could be achieved thermally or *via* Brønsted acid promotion (Scheme 22). Under either condition, these processes are highly regioselective for the α-position, and highly stereoselective for the *E*-configuration. For example, when allenamides with both α- and γ-substitutions were examined, the 1,3-H shift in this case was found to be completely regioselective occurring exclusively from the α-position to afford highly substituted (*E*)-3-amido-trienes 83a–83c in good yields.

**Scheme 23:**
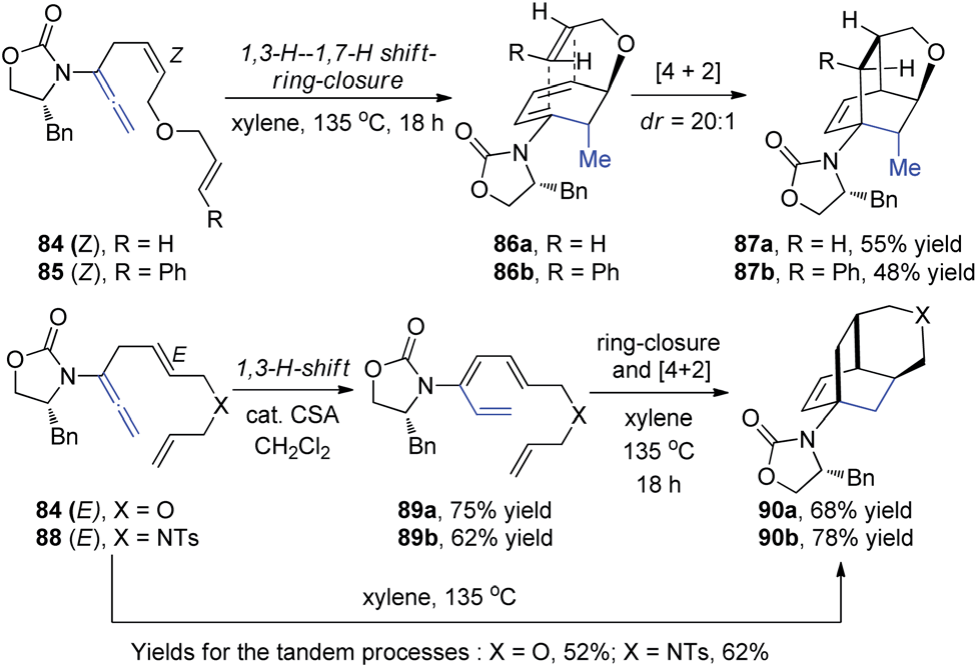
The tandem process of 1,3-H–1,7-H shift-6π-electron pericyclic ring-closure-[4 + 2] cycloaddition.

Hsung *et al.*^30^ then described a new torquoselective ring closure of chiral amide-substituted 1,3,5-hexatrienes and its application in tandem with [4 + 2] cycloaddition. They found that the reactions of allenamides 84-*Z* and 85-*Z* led to tricycles 87a and 87b as single isomers *via* the highly stereoselective [4 + 2] cycloaddition of cyclic amido dienes 86a and 86b, respectively, thereby constituting a quadruple tandem process entailing a 1,3-H-1,7-H shift-6π-electron pericyclic ring-closure-[4 + 2] cycloaddition. In contrast, reactions of allenamides 84-*E* and 88-*E* led to tricycles 90a and 90b in excellent yields and high diastereoselectivity proceeding from amidotrienes 89a and 89b, respectively, or directly from the allenamides in a triple tandem process (Scheme 23). These tandem processes provide a rapid assembly of complex tricycles from very simple allenamides, thereby demonstrating their tremendous power and synthetic potential.

**Scheme 24:**
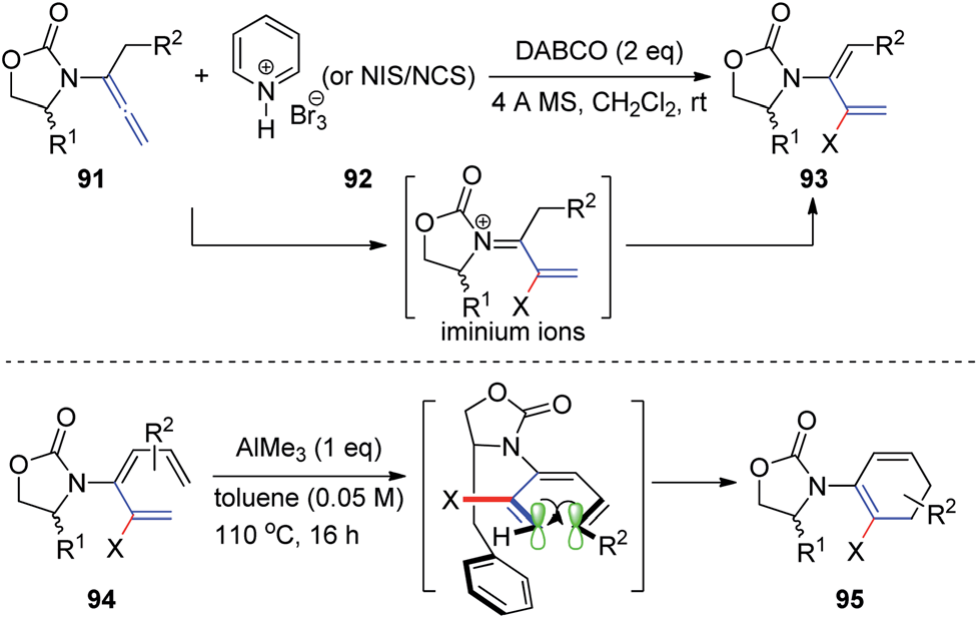
Synthesis of 2-halo-3-amidodi- and -trienes *via* electrophilic halogenation of allenamides.

Subsequently, Hsung^31^ described a synthetic access to rare 2-halo-3-amidodi- and -trienes 93*via* electrophilic halogenation of allenamides 91. These reactions were thought to proceed through the *N*-acyl iminium ion intermediate. Moreover, the successful *de novo* synthesis of chiral 2-halo-3-amidotrienes 94 enabled diastereoselective 6π-electron electrocyclizations *via* a challenging remote 1,6-asymmetric induction with the addition of AlMe_3_. A potential model is presented in Scheme 24 to rationalize the selectivity.

**Scheme 25:**
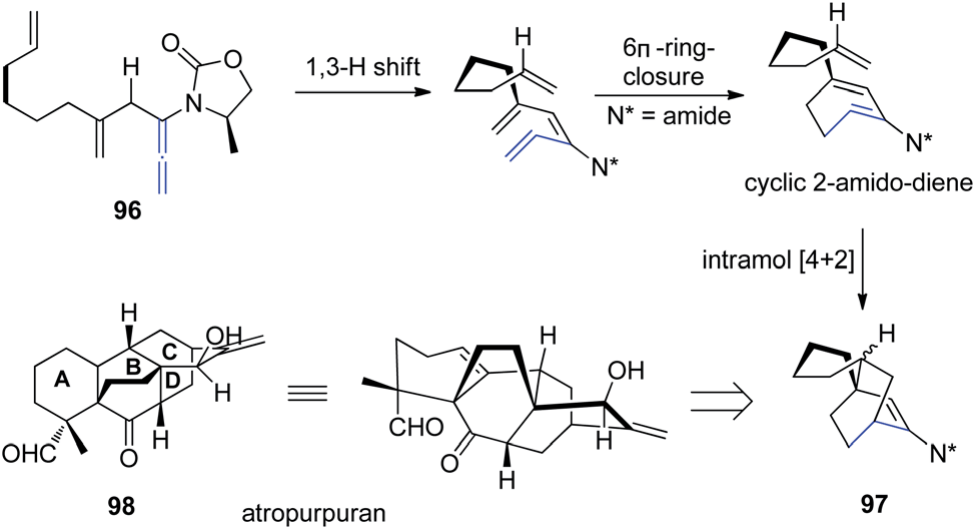
Synthesis of BCD-ring of atropurpuran *via* a 1,3-hydrogen shift, 6π-electron pericyclic ring closure, and an intramolecular Diels–Alder cycloaddition.

Based on a previous report of a highly stereoselective tandem sequence consisting of an allenic 1,3-hydrogen shift of allenamide 96, followed by 6π-electron pericyclic ring closure, and an intramolecular Diels–Alder cycloaddition, Hsung *et al.*^32^ later described an approach toward the BCD-ring of atropurpuran 98 employing this tandem sequence. While the pericyclic ring closure required the assistance of a Lewis acid, the entire process was highly stereoselective for the *endo*-cycloadduct 97 (Scheme 25).

**Scheme 26:**
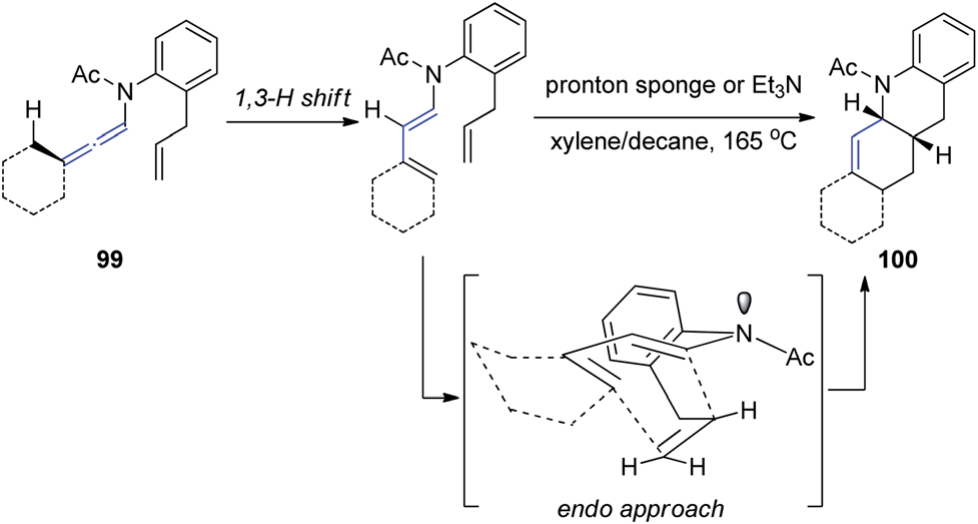
Oppolzer's intramolecular Diels–Alder cycloaddition *via* γ-isomerization of allenamides.

Hsung^33^ further developed a new approach to Oppolzer's intramolecular Diels–Alder cycloaddition (IMDA) through the γ-isomerization of readily available *N*-tethered allenamides 99. These IMDA reactions are performed in tandem with the allenamide isomerization or 1,3-H shift, *via* an *endo*-transition state, as shown in Scheme 26, leading to complex nitrogen heterocycles 100 in a highly stereoselective manner.

**Scheme 27:**
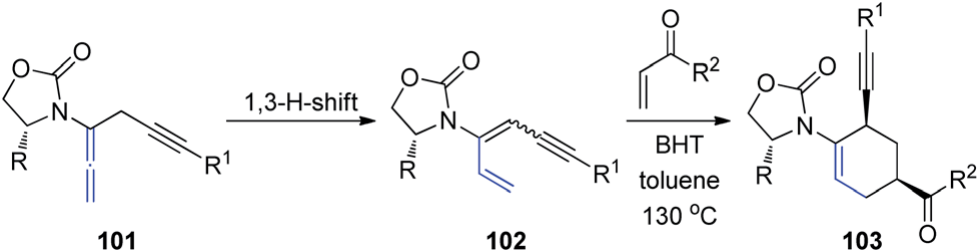
The tandem propargylation-1,3-H-shift sequence of chiral allenamides.

Recently, the same group described a tandem propargylation-1,3-H-shift sequence of chiral allenamides 101 to access both *E* and *Z* isomers of chiral 3-amidodienynes (Scheme 27).^34^ Moreover, the application of (*Z*)-3-amidodienynes 102 in Diels–Alder cycloadditions gave *endo-II* products 103 in good yields and excellent selectivity, while the reactivity of the corresponding (*E*)-3-amidodienynes toward electrocyclization was inadequate.

## 5. Other metal-free allenamide functionalization

**Scheme 28:**
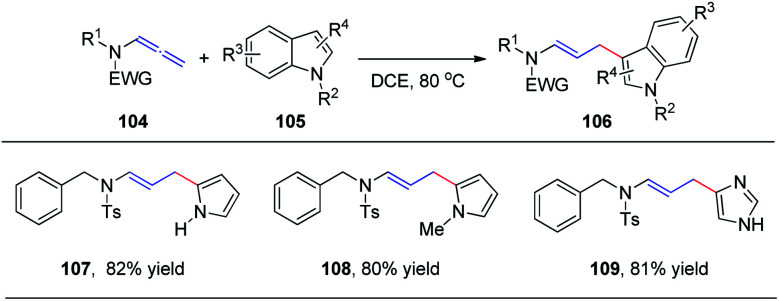
Intermolecular addition of indoles to allenamides under thermal conditions.

In 2015, we reported the first catalyst-free intermolecular addition of indoles 105 to the distal double bond of allenamides 104 (Scheme 28).^35^ The reaction proceeds smoothly at 80 °C to provide a series of (*E*)-enesulfonamide/enamide derivatives 106 in high yields with excellent regioselectivity. Interestingly, pyrrole, methylpyrrole, and imidazole were likewise efficient nucleophiles, affording the desired products 107, 108, and 109 in 82%, 80%, and 81% yields, respectively.

**Scheme 29:**
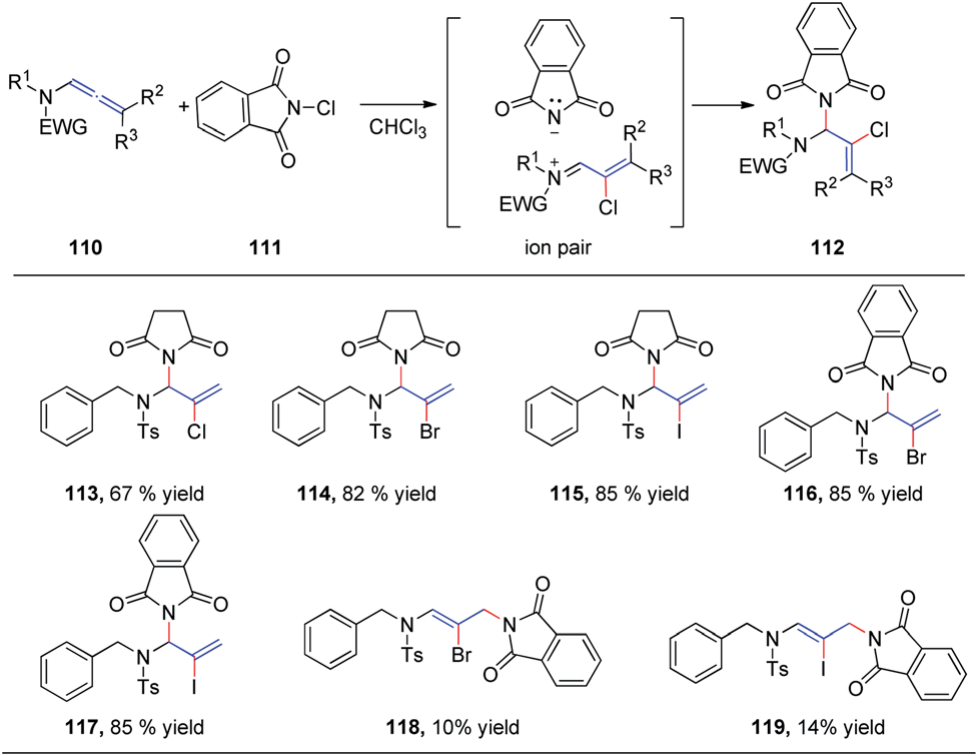
Intermolecular addition of *N*-chlorophthalimide to allenamides.

We subsequently reported the regioselective 1,2-addition of allenamides 110 to *N*-chlorophthalimide 111 for the synthesis of 2-chloro allylic aminal derivatives 112*via* an ion pair composed of a σ-complex and the imide conjugate base (Scheme 29).^36^ This reaction was conducted under very mild conditions and afforded yields of up to 99%. *N*-Haloimides served as both electrophiles and nucleophiles in this reaction. Moreover, *N*-chlorosuccinimide, *N*-bromosuccinimide, *N*-iodosuccinimide, *N*-bromophthalimide, and *N*-iodophthalimide were all efficient substrates for the reaction, affording the desired 1,2-adducts 113–117 in moderate to good yields. In addition, 1,4-adducts, 118 and 119, were also isolated when *N*-bromophthalimide and *N*-iodophthalimide were used as the reactants.

**Scheme 30:**
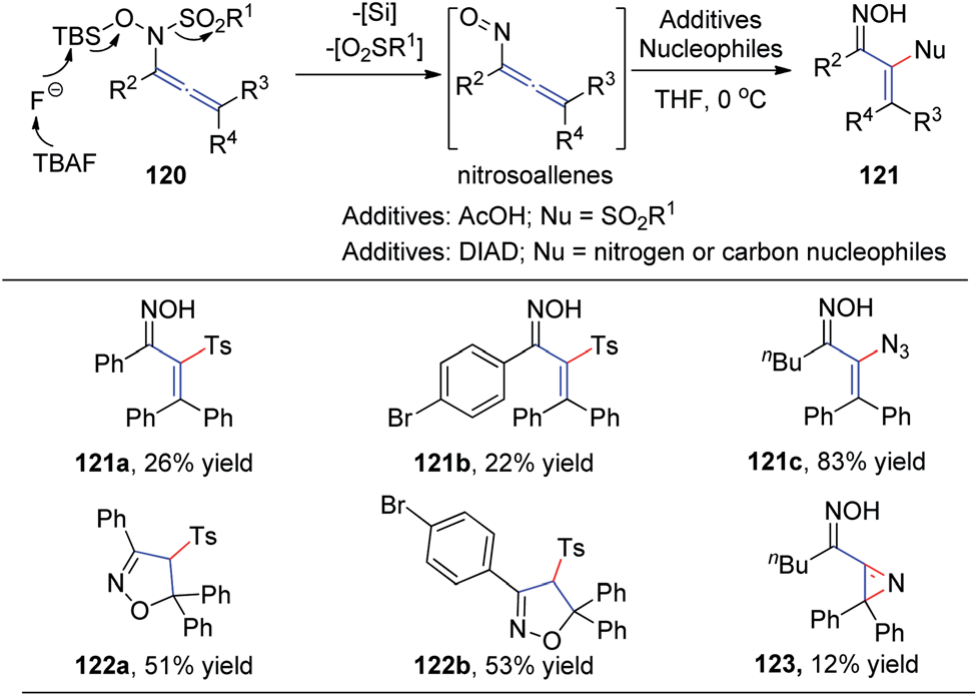
The synthesis of α-functionalized enoximes *via* nucleophilic substitution of nitrosoallenes.

In 2015, Tanimoto *et al.*^37^ developed a synthesis of α-functionalized enoximes 121*via* nucleophilic substitution of nitrosoallenes, a novel chemical species prepared from allenyl *N*-hydroxysulfonamides 120 (Scheme 30). Introduction of various nucleophiles proceeded smoothly to create C–N, C–O, C–S, C–F, and C–C bonds in the presence of azodicarboxylates. Interestingly, α-sulfonyl enoximes 121a and 121b were generated *via* sulfone transfer in the presence of AcOH. Moreover, when all the substituents on the allenylamides were aryl groups, 2-isoxazolines 122a and 122b were afforded as major products, derived from the cyclocondensation of the initially generated vinylsulfones, likely because of their steric bulkiness. Vinyl azide 121c was produced in excellent yield as an inseparable mixture with 2*H*-azirine 123.

**Scheme 31:**
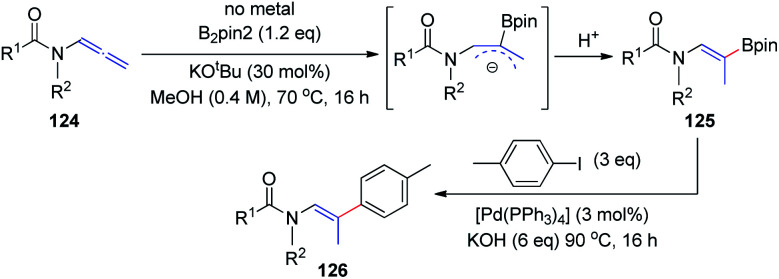
Transition-metal-free borylation of electron-deficient allenamide variants.

In 2018, the Fernández and Vicario group^38^ described a high-yielding transition-metal-free borylation of the distal double bond of electron-deficient variants of allenamides 124, proceeding with complete stereocontrol to provide *Z* isomers exclusively (Scheme 31). The acyl groups on the amine moiety were crucial for obtaining complete stereoselectivity, owing to the formation of a stable allylic anion intermediate, which is further regioselectively protonated to give the final product 125. This transition-metal-free borylation can be followed by Pd-catalyzed cross coupling with aryl iodides, to generate trisubstituted olefins 126 in a stereoselective manner.

## 6. Conclusions

Recent advances in transition-metal-free functionalization of allenamides are summarized herein. These innovative transformations have rendered allenamides highly versatile building blocks in organic synthesis, enabling the assembly of a diverse array of carbo- and heterocyclic structures that can serve as platforms for further transformations. Undoubtedly, the level of interest in allenamides from the synthetic community is immensely high, and there is a tremendous momentum to continue developing allenamide methodology and hence expand their synthetic utility.

## Conflicts of interest

There are no conflicts to declare.

## Supplementary Material

## References

[cit1] Wu X., Gong L. Z. (2019). Synthesis.

[cit2] Hubert A. J., Viehe H. G. (1968). J. Chem. Soc. C.

[cit3] Praveen C. (2019). Coord. Chem. Rev..

[cit4] (a) Asymmetric Brønsted Acid Catalysis, ed. M. Rueping, D. Parmar and E. Sugiono, Wiley-VCH, Weinheim, 2016

[cit5] Tran V. T., Nimmagadda S. K., Liu M. Y., Engle K. M. (2020). Org. Biomol. Chem..

[cit6] Romano C., Jia M. Q., Monari M., Manoni E., Bandini M. (2014). Angew. Chem., Int. Ed..

[cit7] Giacinto P., Bottoni A., Garelli A., Miscione G. P., Bandini M. (2018). ChemCatChem.

[cit8] Manoni E., Gualandi A., Mengozzi L., Bandini M., Cozzi P. G. (2015). RSC Adv..

[cit9] Yang X. Y., Toste F. D. (2016). Chem. Sci..

[cit10] Villar L., Uria U., Martinez J. I., Prieto L., Reyes E., Carrillo L., Vicario J. L. (2017). Angew. Chem., Int. Ed..

[cit11] Yang K., Bao X. Z., Liu S. Y., Xu J. N., Qu J. P., Wang B. M. (2018). Eur. J. Org. Chem..

[cit12] Yang B. M., Zhai X. J., Feng S. B., Hu D. Y., Deng Y. H., Shao Z. H. (2019). Org. Lett..

[cit13] Biswas S., Kim H., Cao K. L., Shin S. (2020). Adv. Synth. Catal..

[cit14] Hummel S., Kirsch S. F. (2011). Beilstein J. Org. Chem..

[cit15] Noguchi M., Okada H., Watanabe M., Okuda K., Nakamura O. (1996). Tetrahedron.

[cit16] Hyland C. J. T., Hegedus L. S. (2006). J. Org. Chem..

[cit17] Zhu Y. X., Yin G. W., Hong D., Lu P., Wang Y. G. (2011). Org. Lett..

[cit18] Yin G. W., Zhu Y. X., Zhang L., Lu P., Wang Y. G. (2011). Org. Lett..

[cit19] Li H. H., Li X. X., Zhao Z. G., Ma T., Sun C. Y., Yang B. W. (2016). Chem. Commun..

[cit20] Li H. H., Li X. X., Zhao Z. G., Lin C. B., Ma T., Sun C. Y., Yang B. W., Fu X. L. (2016). Tetrahedron Lett..

[cit21] Li Y., Luo G. L., Li X. X., Zhao Z. G. (2018). New J. Chem..

[cit22] Yuan X., Man X., Li X. X., Zhao Z. G. (2018). Tetrahedron.

[cit23] Huang R. H., Xu P. Y., Wang W. X., Peng G., Yu H. (2020). Tetrahedron Lett..

[cit24] Luo G. L., Liu Y. C., Ding N., Li X. X., Zhao Z. G. (2019). ACS Omega.

[cit25] Man X., Liu Y. C., Li X. X., Zhao Z. G. (2019). New J. Chem..

[cit26] Chen S. F., Yan Q., Zhao H. Y., Li B. G. (2013). J. Org. Chem..

[cit27] Stille J. L. (1986). Angew. Chem., Int. Ed..

[cit28] Hayashi R., Hsung R. P., Feltenberger J. B., Lohse A. G. (2009). Org. Lett..

[cit29] Hayashi R., Feltenberger J. B., Lohse A. G., Walton M. C., Hsung R. P. (2011). Beilstein J. Org. Chem..

[cit30] Hayashi R., Feltenberger J. B., Hsung R. P. (2010). Org. Lett..

[cit31] Hayashi R., Walton M. C., Hsung R. P., Schwab J. H., Yu X. L. (2010). Org. Lett..

[cit32] Hayashi R., Ma Z. X., Hsung R. P. (2012). Org. Lett..

[cit33] Feltenberger J. B., Hsung R. P. (2011). Org. Lett..

[cit34] Ma Z. X., Fang L. C., Haugen B. J., Bruckbauer D., Feltenberger J. B., Hsung R. P. (2017). Synlett.

[cit35] Li H. H., Ma T., Li X. X., Zhao Z. G. (2015). RSC Adv..

[cit36] Li H. H., Li X. X., Zhao Z. G., Yuan X., Sun C. Y. (2017). Org. Biomol. Chem..

[cit37] Tanimoto H., Yokoyama K., Mizutani Y., Shitaoka T., Morimoto T., Nishiyama Y., Kakiuchi K. (2016). J. Org. Chem..

[cit38] Garcia L., Sendra J., Miralles N., Reyes E., Carbo J. J., Vicario J. L., Fernández E. (2018). Chem.–Eur. J..

